# Fabrication of Fluorinated Magnetic Covalent Organic Frameworks for the Extraction of Fluoroquinolone Residues Coupled with HPLC Detection

**DOI:** 10.3390/molecules31061025

**Published:** 2026-03-19

**Authors:** Jichao Liu, Xiuzhuang Li, Jiaojiao Yu

**Affiliations:** College of Petrochemical Engineering, Lanzhou Petrochemical University of Vocational Technology, Lanzhou 730060, China

**Keywords:** fluorinated magnetic covalent organic framework, magnetic solid phase extraction, fluoroquinolones, high performance liquid chromatograph

## Abstract

Fluoroquinolones (FQs) are a kind of antibiotics, which are widely used in animal husbandry and aquaculture. However, the abuse of FQs can result in residues in foodstuffs of animal origin. Therefore, it is essential to establish a sensitive and accurate detection method for determination of FQs in food samples. An effective sample pretreatment method is a crucial procedure for enrichment of trace target compounds from complex matrix before HPLC analysis. As an emerging kind of sample pretreatment methods, magnetic solid-phase extraction (MSPE) has attracted much interest due to its characteristics including low cost, simplicity, and rapidity. In this study, a novel fluorinated magnetic covalent organic framework (Fe_3_O_4_@PDA@COF) was fabricated, which was used as an adsorbent in MSPE as well as coupled with HPLC to determine FQs in food samples. Under optimal conditions, the developed Fe_3_O_4_@PDA@COF-MSPE-HPLC-UV method possessed a wide linear range (1–250 µg·kg^−1^) and low limits of detection (0.5–0.7 µg·kg^−1^) with good linearity (R^2^ ≥ 0.9938). Additionally, the method has been used to adsorb FQs from chicken samples. The recoveries of target FQs in spiked samples were 84.2–106.7% with relative standard deviations (RSDs) below 7.8%. These results demonstrated that the established method provides an efficient and sensitive solution for monitoring FQ residues in foodstuffs.

## 1. Introduction

Fluoroquinolones (FQs) are a kind of amphiphilic synthetic antibiotics. Owing to their high efficacy and broad-spectrum antibacterial activity, FQs are extensively employed in animal husbandry and aquaculture for disease prevention and treatment [1]. Nevertheless, the massive use of FQs may lead to residues in animal-derived foods, which thereby affect the health of humans, encompassing allergic reactions, hepatotoxicity, and pathogen resistance [2,3]. Consequently, it is essential to ensure human health and food safety that accurately determines FQs in meat matrices.

At present, FQs are usually analyzed by chromatographic methods, such as high-performance liquid chromatography (HPLC) [4], liquid chromatography-tandem mass spectrometry (LC-MS/MS) [5], etc. Among these, HPLC is an indispensable method for the analysis of FQs because of its outstanding separation efficiency and accuracy [6]. However, it is difficult to directly analyze by using HPLC due to low concentrations of FQs and complex matrix. To ensure accurate analysis given the low analyte concentration and complex matrix, a sample pretreatment procedure is essential to separate and extract the trace target compounds.

Up to now, a variety of sample pretreatment methods has been used for FQ extraction in different matrix, for instance, solid-phase extraction (SPE) [7], dispersive solid-phase extraction (DSPE) [8], magnetic solid-phase extraction (MSPE) [9], the QuEChERS [10] and pipette-tip solid-phase extraction (PT-SPE) [11]. Among those, MSPE has been attracting extensive attention because of its merits such as low cost, simplicity, and rapidity [12]. In MSPE, the adsorbent serves as the core of the extraction process. Despite extensive research on adsorbents, the lack of adsorption specificity remains the principal challenge for adsorbents. Consequently, designing adsorbents with high selectivity and capacity for target analytes in complex matrices remains challenging.

Covalent organic frameworks (COFs) are an emerging kind of porous materials, which have demonstrated outstanding advantages such as low density, chemical stability, high surface areas and adjustable pore size [13,14]. Therefore, COF has great application potential as an adsorbent in the field of sample pretreatment [15,16,17,18]. It has been demonstrated that the extraction performance of COFs toward fluorine-containing analytes can be significantly enhanced through incorporating fluorine atoms into COF [19,20]. Fluorinated COFs have been applied to enrich benzoylurea insecticides [21], fipronils [22], perfluorinated compounds [23] as well as tetrabromobisphenol A [24], etc. Given the fluorine-containing nature of FQs, we infer that the fluorinated COF could serve as a potential adsorbent for these compounds.

**Figure 1:**
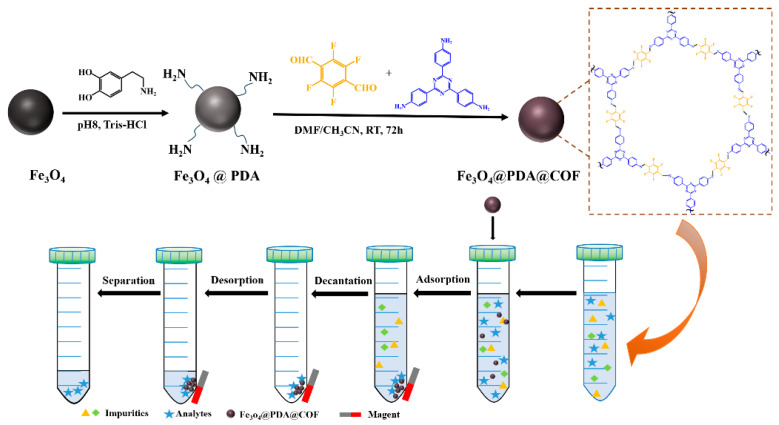
Schematic fabrication procedure of Fe_3_O_4_@PDA@COF and the application to MSPE process.

Herein, a fluorinated magnetic COF (Fe_3_O_4_@PDA@COF) was prepared by polydopamine (PDA) grafting Fe_3_O_4_ nanoparticles as magnetic core and COF as shell for MSPE of FQs residue (Figure 1). In this study, lomefloxacin hydrochloride (LOM), sparfloxacin (SPX), and marbofloxacin (MFB) were selected as the target analytes (Figure S1). The COF was fabricated by 2,3,5,6-tetrafluoroterephthaldehyde (TFTA) and 1,3,5-tris(4-aminophenyl)triazine (TAPT) as two monomers. PDA as a bridge grafting on the surface of Fe_3_O_4_ nanoparticles promoted the growth of COF. It is worth mentioning that the Fe_3_O_4_@PDA@COF exhibits effective extraction of FQs by the aid of fluorophilic interaction owing to the introduction of fluorine atom. Furthermore, factors that potentially affect the MSPE efficiency were optimized, and the adsorption feature of FQs in the Fe_3_O_4_@PDA@COF was investigated by adsorption kinetics. Finally, a simple and sensitive method for the determination of FQs in meat samples by combining Fe_3_O_4_@PDA@COF-based MSPE with HPLC was established.

## 2. Results

### 2.1. Characterization of Fe_3_O_4_@PDA@COF

**Figure 2:**
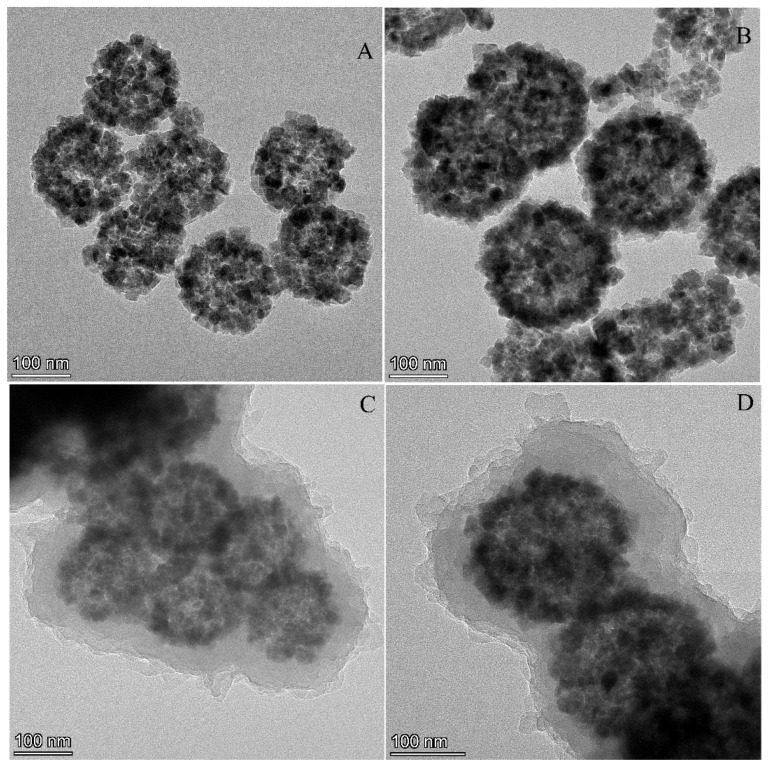
TEM images of Fe_3_O_4_ (**A**), Fe_3_O_4_@PDA (**B**), Fe_3_O_4_@PDA@COF (**C**,**D**).

The morphology of Fe_3_O_4_, Fe_3_O_4_@PDA and Fe_3_O_4_@PDA@COF was characterized by TEM. As could be seen from Figure 2A, Fe_3_O_4_ had a spherical shape, and the size was 120–140 nm. As shown in Figure 2B, compared with unmodified Fe_3_O_4_, a thin dark layer can be observed around the periphery of Fe_3_O_4_@PDA nanospheres, indicating that the PDA shell was successfully coated onto the Fe_3_O_4_ core. The size of Fe_3_O_4_@PDA increases to 160–170 nm. Figure 2C,D showed that Fe_3_O_4_@PDA@COF exhibits a distinct core–shell structure with the size exceeding 200 nm, while the COF shell is approximately 30 to 60 nm. These results suggested the successful encapsulation of the COF shell layer on the Fe_3_O_4_@PDA surface.

**Figure 3:**
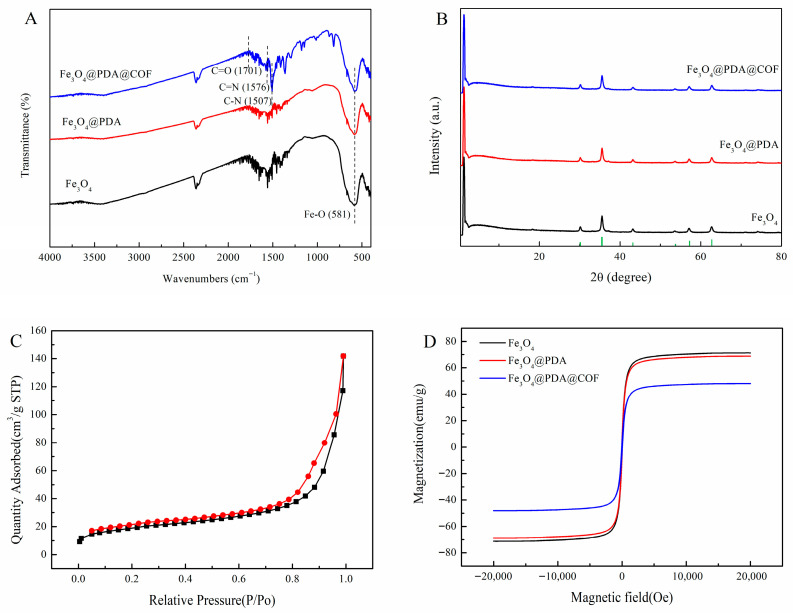
FT-IR spectra (**A**), XRD patterns (**B**) (the green vertical bars represent the standard Fe_3_O_4_ pattern (JCPDS No. 19-0629)), N_2_ adsorption (black color) and desorption (red color) isotherms of Fe_3_O_4_@PDA@COF (**C**), and magnetic hysteresis curves (**D**).

The synthesized Fe_3_O_4_, Fe_3_O_4_@PDA and Fe_3_O_4_@PDA@COF were further characterized using FT-IR spectroscopy. As shown in Figure 3A, the strong peak appeared at approximately 581 cm^−1^ in the spectra of Fe_3_O_4_, Fe_3_O_4_@PDA and Fe_3_O_4_@PDA@COF, which is attributed to the Fe-O stretching vibration [25]. The characteristic peak observed at 1507 cm^−1^ can be assigned to the C–N vibration of the triazine ring [26]. The appearance of typical imine bond absorption at 1576 cm^−1^ confirms the successful formation of the imine-linked COF shell [27]. A weak signal around 1701 cm^−1^ is also observed, which may originate from residual carbonyl groups in the PDA layer or unreacted terminal aldehydes. A weak band observed in the 2500–2250 cm^−1^ region is attributed to atmospheric CO_2_, a common artifact in FTIR measurements using the KBr pellet method, and does not correspond to any functional groups in the synthesized materials.

The crystalline structures of Fe_3_O_4_, Fe_3_O_4_@PDA and Fe_3_O_4_@PDA@COF were examined by XRD. The result was demonstrated in Figure 3B. All diffraction peaks of synthesized Fe_3_O_4_ matched well with the standard Fe_3_O_4_ pattern (JCPDS No. 19-0629), with characteristic peaks at 2θ = 30.1°, 35.5°, 43.1°, 53.4°, 57.0°, and 62.6° corresponding to the (220), (311), (400), (422), (511), and (440) planes, respectively [28]. Additionally, as shown in Figure S2, diffraction peaks at 2.9°, 5.7°, and 7.4° was observed, which can be attributed to the (100), (200), and (210) facets of COF based on TAPT-TFTA, respectively. This is basically consistent with the results in the literature [26]. The results showed that Fe_3_O_4_@PDA@COF was successfully synthesized with good crystallinity.

N_2_ adsorption–desorption experiments were carried out to evaluate the pore size distribution and specific surface area. Figure 3C showed that the Brunauer–Emmett–Teller (BET) surface area of Fe_3_O_4_@PDA@COF was 67.8 m^2^·g^−1^. Barrett–Joyner–Halenda (BJH) analysis of the adsorption data indicated that the pore size of Fe_3_O_4_@PDA@COF was mainly concentrated at 16.9 nm.

The magnetic properties of three materials were evaluated by measuring magnetic hysteresis curves. As shown in Figure 3D, the saturated magnetization of Fe_3_O_4_, Fe_3_O_4_@PDA and Fe_3_O_4_@PDA@COF reached 71.2, 68.7 and 48.1 emu·g^−1^, respectively. Although the formation of the COF shells led to a slight decrease in magnetism, Fe_3_O_4_@PDA@COF retained sufficiently high saturation magnetization to enable effective magnetic separation.

### 2.2. Optimization of the MSPE Conditions

To optimize the adsorption of FQs, various parameters including adsorbent dosage, adsorption time, the solution pH, eluent and desorption time were studied with recovery as the main indicator.

**Figure 4:**
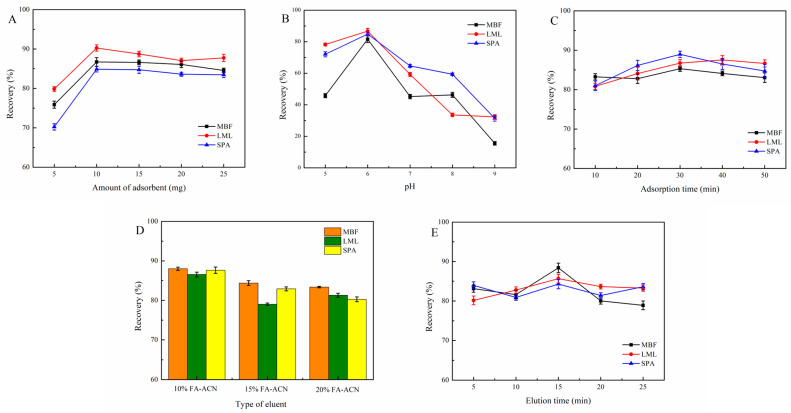
Influence of adsorbent dosage (**A**), sample pH (**B**), adsorption time (**C**), type of eluent (**D**), and desorption time (**E**) on the recovery of FQs.

Since the adsorbent dosage affects extraction efficiency directly, the effect of adsorbent dosage (5–25 mg) was evaluated. As illustrated in Figure 4A, the recovery of FQs increased with increasing dosage from 5 mg to 10 mg and then plateaued. Therefore, 10 mg Fe_3_O_4_@PDA@COF was selected as the optimal dosage.

The solution pH is an important parameter that significantly governs MSPE efficiency. As amphiphilic compounds with pKa between 5.66 and 8.56, FQs take different forms at different pHs [29,30], as depicted in Figure 4B, the recovery of FQs increased with increasing pH from 5 to 6 but decreased with increasing pH from 6 to 9. This trend can be attributed to speciation changes in the FQs. At pH 6, FQs predominantly exist in neutral molecular form, which favors adsorption via π-π and hydrophobic interactions with the adsorbent. At lower pH, the protonated FQs display charge-charge repulsion with COF shell, reducing adsorption efficacy. When the solution pH exceeds 6, the FQs become deprotonated and acquire negative charges, resulting in repulsive interactions between FQs and COF shell, thereby reducing adsorption. Based on these results, pH 6 was chosen as the optimal condition.

In order to optimize the extraction efficiency, adsorption time (i.e., 10, 20, 30, 40, and 50 min) was evaluated. Figure 4C shows that the adsorption time increases from 10 min to 30 min; the extraction efficiency significantly improves and tends to stabilize at 30 min. Thus, 30 min was selected as the optimal adsorption time.

In order to study the influence of eluent type, MeOH, ACN, 10% formic acid-acetonitrile (10% FA-ACN), 15% formic acid-acetonitrile (15% FA-ACN) and 20% formic acid-acetonitrile (20% FA-ACN) were applied as the eluents. It should be noted that the eluent is used in the MSPE process to desorb analytes from the adsorbent. As illustrated in Figure 4D, the maximum desorption was achieved by using 10% FA-ACN as the eluent. It may be due to the protonation of FQs under acidic conditions, which converts them into ionic state, reducing its affinity to the adsorbent and facilitating efficient desorption. Although COFs generally exhibit good chemical stability, prolonged exposure to highly acidic conditions (such as 15% FA–ACN and 20% FA–ACN) may partially damage the imine linkages or other bonds within the COF structure. This could lead to pore collapse or alter the surface properties of the Fe_3_O_4_@PDA@COF adsorbent, thereby reducing desorption efficiency. Consequently, 10% FA-ACN was selected as the optimal eluent.

The desorption time is a critical parameter for ensuring the complete desorption of FQs from Fe_3_O_4_@PDA@COF. As shown in Figure 4E, the recovery increased from 5 to 15 min, and no significant change was observed when ultrasound treatment exceeded 15 min. Thus, 15 min was elected as the optimal desorption time.

The reusability of adsorbents is critical for application in MSPE. Thus, the extraction performance of Fe_3_O_4_@PDA@COF was assessed over multiple adsorption–desorption cycles. As shown in Figure S3, the recovery of the FQs remained stable with no significant decline during the four cycles, indicating good reusability of the prepared adsorbent. However, after eight cycles, the recovery of three FQs decreased substantially to 42.7–54.6%, suggesting that Fe_3_O_4_@PDA@COF maintains satisfactory performance for four regeneration cycles.

### 2.3. Evaluation the Adsorption Performances of Fe_3_O_4_@PDA@COF

Adsorption capacity is a crucial parameter in the theory of adsorption equilibrium and adsorption rate. Additionally, adsorption kinetics elucidates the dependence of the adsorption process on time, focusing on the adsorption rate and adsorption dynamic equilibrium.

The kinetic adsorption behavior was evaluated applying the pseudo-first-order (Equation (1)) and pseudo-second-order (Equation (2)) models, respectively [31].

(1) $$\ln\left(Q_{e}-Q_{t}\right)=lnQ_{e}-k_{1}t$$

(2) $$\frac{t}{Q_{t}}=\frac{1}{k_{2}Q_{e}^{2}}+\frac{1}{Q_{e}}t$$

where *Q_t_* and *Q_e_* denote the adsorption capacity (per unit mass of the adsorbent) at time (*t*) and at equilibrium, respectively. Additionally, *k*_1_ and *k*_2_ denote the rate constant of pseudo-first-order and pseudo-second-order kinetics, respectively.

**Figure 5:**
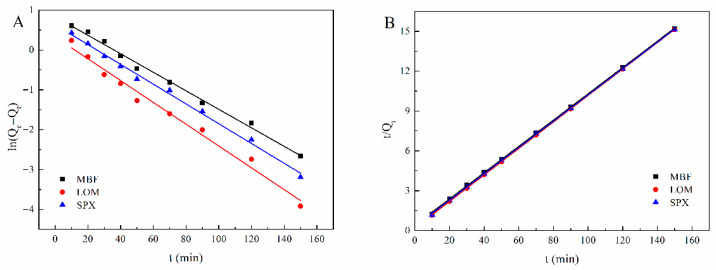
Fitting of pseudo-first order (**A**) and pseudo-second order (**B**) kinetic model of FQs on Fe_3_O_4_@PDA@COF.

The linear fitting results for both kinetic models are presented in Figure 5, with the corresponding parameters summarized in Table 1. The obtained results showed that the pseudo-second-order model (R^2^ = 0.9998–1) provided a significantly better fit than the pseudo-first-order model (R^2^ = 0.9856–0.9952), while the experimentally obtained *Q_e_* is closer to the expected *Q_e_* of the pseudo-second-order model, suggesting that the adsorption process between Fe_3_O_4_@PDA@COF and FQs aligns more closely with pseudo-second-order kinetics, indicative of chemical interaction [32].

### 2.4. Method Validation and Sample Analysis

To evaluate the analytical performance of the developed MSPE-HPLC-UV method for the determination of FQs in chicken samples, the method was validated in terms of linearity, sensitivity, precision, and accuracy. The results are shown in Table 2. Under optimal experimental conditions, the correlation coefficients (R^2^) of >0.99 indicated good linearity for the three FQs within the range of 1 to 250 μg·kg^−1^. The limits of detection (LODs) and quantification (LOQs) for the three FQs, calculated at signal-to-noise (S/N) ratios of 3 and 10 using spiked sample chromatograms were in the range of 0.5–0.7 μg·kg^−1^ and 1.7–2.5 μg·kg^−1^, respectively. The range of inter-day and intra-day RSDs (*n* = 3) of the developed MSPE method was 3.9–6.1% and 5.4–7.8%, respectively. These results revealed that the proposed method had good reproducibility and repeatability, with RSD well within the acceptable range (<20%) recommended by the SANTE/11813/2017 [33] guideline for residue analysis in food matrices.

**Figure 6:**
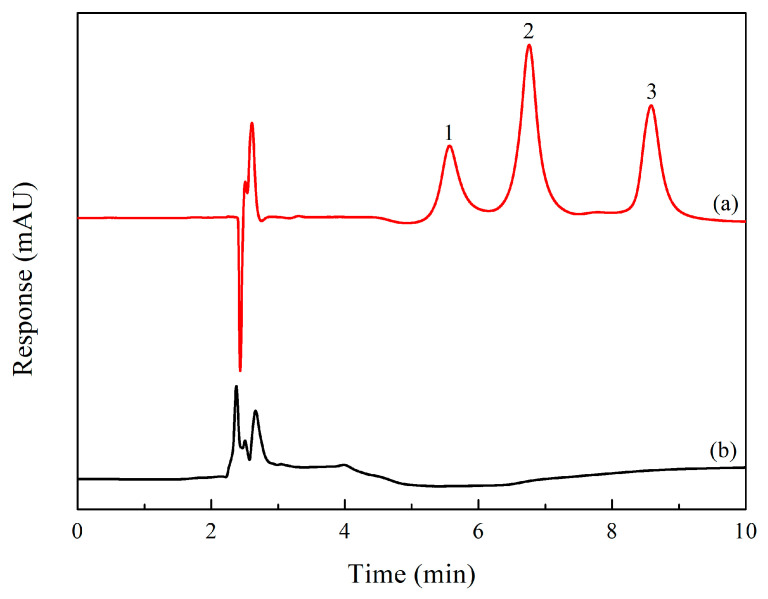
Chromatograms obtained after MSPE under optimized conditions: (**a**) sample spiked with 1 µg·mL^−1^ of FQ standards and blank sample (**b**). Peaks: 1, MBF; 2, LOM; 3, SPX.

In order to assess the practicality of the proposed MSPE-HPLC-UV method for FQ determination, chicken samples were analyzed. Calibration curves were constructed by spiking blank chicken extracts with FQs over the range of 1–250 μg·kg^−1^, yielding correlation coefficients (R^2^) > 0.99. Recovery experiments were conducted at three representative concentrations (5, 50, and 100 μg·kg^−1^) to assess method accuracy. Table 3 showed that no FQs were detected in the analyzed chicken samples. Since no FQ standards were detected in the blank samples, accuracy was evaluated by recovery experiments using spiked samples. The recovery of FQs ranged from 84.2% to 106.7%, with RSD < 7.8%. The recoveries (84.2–106.7%) were within the acceptable range (70–120%) specified by international guidelines for trace analysis in complex food matrices [SANTE/11813/2017] [33], demonstrating that the established MSPE-HPLC-UV method is reliable for detecting FQs in chicken samples. The chromatograms of the standard solution and the actual samples are exhibited in Figure 6.

### 2.5. Comparison with Other Methods

A systematic comparison with previously reported methods was conducted to evaluate the performance of the developed approach. As shown in Table 4, the present method offers excellent recovery, simplicity, and efficiency. Although the established MSPE-HPLC-UV method was not the most sensitive among the other methods compared in terms of detection limit, it still has recommendable advantages. Compared with SPE, the developed method based on MSPE enables rapid separation simply by applying an external magnet. This not only significantly shortens the total sample processing time but also drastically reduces the consumption of organic solvents. In addition, the developed MSPE-HPLC-UV method not only utilizes a cost-efficient UV detector and less-complicated flows but also achieves satisfactory extraction efficiency alongside a significantly reduced total analysis time. These attributes make it an economically efficient solution for routine FQ analysis in meat samples. Reliable performance is especially crucial in the absence of ultratrace detection.

## 3. Discussion

FQs are widely used for the prevention and treatment of various animal diseases. However, the massive use of FQs may lead to residues in animal-derived foods, which thereby affect the health of humans. It is significant to establish a sensitive method for detection of trace FQs in meat samples. In this work, a novel fluorinated magnetic adsorbent Fe_3_O_4_@PDA@COF was developed to extract FQs combined with HPLC for the determination of FQs in meat samples.

In terms of morphology, the fabricated Fe_3_O_4_@PDA@COF had a core–shell structure with a size exceeding 200 nm, where the Fe_3_O_4_ core size is 120–140 nm and the COF shell size is 30–60 nm. An excessively thin layer limits adsorption sites, while an overly thick one hampers magnetic responsiveness. Given the Fe_3_O_4_ core size of 120–140 nm, the 30–60 nm shell thickness is considered appropriate because it provides sufficient surface area for abundant adsorption sites while still allowing for rapid magnetic separation. Meanwhile, the imine COF shell with TAPT and TFTA as organic ligands was formed due to the occurrence of imine bond absorption at 1576 cm^−1^ and a C=O stretch band around 1701 cm^−1^ by FT-IR, as well the diffraction peaks of Fe_3_O_4_ and COF by XRD. With a BET surface area of 67.8 m^2^·g^−1^ and a predominant pore size of 16.9 nm, Fe_3_O_4_@PDA@COF had structural features conducive to the physical adsorption of FQs. Additionally, Fe_3_O_4_@PDA@COF retained sufficiently high saturation magnetization to enable effective magnetic separation.

It can be seen from optimal conditions for FQs that solution pH and eluent are significant factors that affect recovery of FQs. The efficiency of both adsorption and desorption is governed by the speciation of FQs. At a solution pH of 6, FQs mainly exist in neutral molecular form, which facilitates adsorption through π-π and hydrophobic interactions with the adsorbent. The protonated FQs at lower pH or deprotonated FQs at higher pH demonstrated charge-charge repulsion between FQs and adsorbents, reducing adsorption efficacy. In terms of elution, lower pH protonated FQs, reducing adsorbent affinity and facilitating elution.

From the results of adsorption kinetics, the adsorption process followed a pseudo-second-order model, suggesting that the chemisorption plays a significant role in the adsorption of FQs onto Fe_3_O_4_@PDA@COF. Based on the structural characteristics of the adsorbent and the target analytes, several synergistic effects may facilitate adsorption. First, the porous structure of Fe_3_O_4_@PDA@COF facilitates the entry and effective adsorption of FQs. Second, the benzene rings in Fe_3_O_4_@PDA@COF are likely to interact with those in FQs by π-π and hydrophobic interactions, as supported by previous reports [37]. Third, given the presence of fluorine in both the Fe_3_O_4_@PDA@COF and the FQs, F-O and F-π interactions may also contribute [38], consistent with the fluorophilic nature [21]. These complementary mechanisms collectively contribute to the rapid and selective adsorption of FQs onto Fe_3_O_4_@PDA@COF. The developed MSPE-HPLC-UV method based on Fe_3_O_4_@PDA@COF was reliable and sensitive with a wide linear range and low limits of detection. The spiked experiments also demonstrated excellent accuracy in complex food matrices. In this study, the established method can not only be used to determine chicken samples but can also be extended to more meat samples.

## 4. Materials and Methods

### 4.1. Chemicals and Reagents

Standards of lomefloxacin hydrochloride (LOM), sparfloxacin (SPX) and marbofloxacin (MFB) were brought from Yuanye Biotechnology Co., Ltd. (Shanghai, China). 4,4′,4″-(1,3,5-Triazine-2,4,6-triyl)trianiline (TAPT), 2,3,5,6-Tetrafluorotelephtal aldehyde (TFTA) were obtained from Macklin Biochemical Co., Ltd. (Shanghai, China). HPLC-grade acetonitrile (ACN) and methanol (MeOH) were purchased from Merck KGaA (Darmstadt, Germany). Ferric chloride hexahydrate (FeCl_3_·6H_2_O) and sodium citrate dihydrate (C_6_H_5_O_7_Na_3_·2H_2_O) were brought from Sinopharm Chemical Reagent Co., Ltd. (Shanghai, China). Urea, dopamine hydrochloride and polyacrylamie (PAM) were brought from Yuanye Biotechnology Co., Ltd. (Shanghai, China). Tris-HCl Buffer (pH 8.5), CH_3_CO_2_H (AcOH), formic acid (FA) and N,N-dimethylformamide (DMF) were obtained from Aladdin Biochemical Technology Co., Ltd. (Shanghai, China). LOM, SPX and MFB standard stock solutions (1 mg·mL^−1^) were prepared by MeOH with 2% formic acid and stored at 4 °C in the dark. Working solutions were obtained by gradually diluting the stock solutions with MeOH.

### 4.2. Characterization

The separation and detection of target FQs were performed using a HPLC (UV230II, Elite, Dalian, China) with a UV detector. X-ray diffraction (XRD) patterns were obtained on a SmartLab SE X-ray diffractometer (Rigaku, Akishima, Tokyo, Japan) with Cu Kα radiation. Fourier transform infrared spectroscopy (FT-IR) spectra were observed on a Nicolet iS5 spectrophotometer (Thermo Fisher, Waltham, MA, USA) using the KBr pellet method. The surface morphologies of the COFs observed by a transmission electron microscopy (TEM) were measured on a Talos F200X transmission electron microscope (FEI, Hillsboro, OR, USA). N_2_ adsorption–desorption curves were recorded on an ASAP 2460 volumetric sorption analyzer (Micromeritics, Norcross, GA, USA). Magnetic hysteresis loops were measured on a 7404 vibrating sample magnetometer (VSM) (LakeShore, Westerville, OH, USA). Detailed parameters for these characterization methods are provided in the Supplementary Materials (Section S1).

### 4.3. Synthesis of Fe_3_O_4_ Nanoparticles

Fe_3_O_4_ nanoparticles were synthesized based on a previously reported method with slight modifications [39]. Typically, 2 mmol of FeCl_3_·6H_2_O, 4 mmol of sodium citrate dihydrate and 6 mmol of urea were separately dissolved in 40 mL of deionized water in one breaker. The mixture was stirred for 1 h and transferred to a Teflon-lined stainless-steel autoclave. Following the reacting at 200 °C for 10 h, the black product was isolated by magnetic separation, thoroughly washed with water and ethanol sequentially, and then dried at 70 °C overnight to yield Fe_3_O_4_ nanoparticles.

### 4.4. Synthesis of Fe_3_O_4_@PDA and Fe_3_O_4_@PDA@COF

Fe_3_O_4_@PDA was prepared by the mussel-inspired functionalization method [40]. Typically, 100 mg of Fe_3_O_4_ nanoparticles was added into 100 mL of 2 mg·mL^−1^ dopamine Tris-HCl buffer with mechanically stirring for 12 h at room temperature. After the 12 h reaction, the product was repeatedly washed with deionized water and ethanol, and then collected magnetic separation. The obtained nanospheres were dried at 60 °C for 24 h.

The Fe_3_O_4_@PDA@COF was synthesized using Fe_3_O_4_ as the magnetic core, PDA as bridge agent, and TAPT and TFTA as the ligands. In the synthesis, 30 mg Fe_3_O_4_ nanoparticle, 61.8 mg of TFTA and 70.9 mg TAPT were dissolved in 4 mL DMF in a 50 mL centrifuge tube, followed by addition of 24 mL ACN and 2 mL aqueous AcOH solution (12 mol·L^−1^), respectively [27]. The resulting mixture was shaken for 30 s and then left to stand undisturbed at 30 °C for 72 h. Subsequently, the precipitate was collected by magnet, washed with ACN, and dried at 60 °C overnight.

### 4.5. MSPE Procedures

A typical MSPE is shown in Figure 1. A total of 10 mg of Fe_3_O_4_@PDA@COF was added to 10 mL standard or sample solution. The mixture was vortexed for 30 min for extraction of target analytes. Following adsorption, the adsorbents were separated by an external magnet. After discarding the supernatant, the adsorbents were eluted with 1 mL 10% formic acid-acetonitrile under ultrasound for 15 min. Finally, the solution was filtered through 0.22 μm membrane for HPLC analysis.

HPLC analysis was conducted using a system equipped with a UV detector with a wavelength of 280 nm. The separation was performed on a C18 column (150 mm × 4.6 mm, 5.0 μm) by using MeOH (A) and 0.5% FA-H_2_O (B) as the mobile phase. The gradient elution was set as follows: 0 min, 30% A; 0–2 min, 43% A; 2–6 min, 45% A; and 6–8 min, 30% A. The injection volume was 20 μL, and the flow rate was 1.0 mL·min^−1^.

Fresh chicken samples were purchased from a local market in Xigu District, Lanzhou, China. Approximately 100 g of chicken breast muscle samples were transported to the laboratory and processed within 5 h of purchase. The prepared chicken sample solutions were stored at 4 °C until analysis. The preparation of sample solutions was carried out on the basis of a reported method with partial modifications [31]. The amount of 2 g of the homogenized chicken sample was weighed into a 50 mL centrifuge tube, followed by spiking with the target FQs. After 10 min of ultrasonicating, 10 mL 0.1 M sodium phosphate buffer (pH 7.0) was added, as well the mixture was ultrasonicated for 15 min. The supernatant was collected. The extraction procedure was repeated twice to ensure comprehensive recovery of FQs from chicken samples. The pooled supernatant was dried using a vacuum rotary evaporator at 40 °C. Then, the residue was reconstituted with 10 mL of deionized water. The obtained solution was filtered (0.22 μm microfiltration membrane) for subsequent MSPE.

### 4.6. Investigation of the Adsorptive Properties of Fe_3_O_4_@PDA@COF

Adsorption characteristics of Fe_3_O_4_@PDA@COF was explored using various FQs including LOM, SPX and MFB as target analytes. A total of 10 mg of Fe_3_O_4_@PDA@COF was introduced to a 15 mL centrifuge tube containing 10 mL of the FQ solution at a concentration of 10 μg⋅mL^−1^. The solution was shaken ranging from 1 to 150 min. After shaking, the supernatant was obtained by magnetic separation using external magnets further filtering (0.22 µm microfiltration membrane). HPLC-UV analysis was employed to determine the equilibrium concentrations. The equilibrium adsorption capacity of Fe_3_O_4_@PDA@COF for FQs can be obtained by Equation (3) [41].

(3) $$Q_{e}=\frac{(c_{0}-c_{1})V}{m}$$

where *Q_e_* (mg⋅g^−1^) represents the equilibrium adsorption capacity. *c*_0_ (μg⋅mL^−1^) and *c*_1_ (μg⋅mL^−1^) signify the initial concentration and the supernatant concentration, respectively. *V* (mL) represents the volume of the FQs solution; *m* (mg) signifies the mass of the Fe_3_O_4_@PDA@COF.

## 5. Conclusions

In summary, this work presented a sensitive MSPE-HPLC method for the FQ detection using a magnetic fluorinated Fe_3_O_4_@PDA@COF, which serves as an adsorbent. The fluorinated Fe_3_O_4_@PDA@COF adsorbent demonstrates superior extraction efficiency because with introduction of F atom in the fluorinated adsorbents, it enables it to adsorb FQs through F-F affinity interaction, combined with π-π and hydrophobic interactions between its abundant benzene rings and the benzene rings of the FQs. The developed method demonstrated excellent analytical performance, high precision (≤7.8%), and low LODs (0.5–0.7 μg·kg^−1^). From the results of adsorption kinetics, the adsorption process followed a pseudo-second-order model, indicating a chemical interaction between Fe_3_O_4_@PDA@COF and FQs. This work develops a cost-effective solution for the determination of FQs in meat; it also offers more choices for the sample pretreatment of antibiotic pollutant residues in complex matrix. While this study focused on method validation for trace analysis, future work could investigate the adsorption isotherms and maximum capacity of Fe_3_O_4_@PDA@COF to further elucidate its thermodynamic properties and potential for high-concentration applications.

## Figures and Tables

**Table 1:** Kinetic parameters for adsorption of FQs onto Fe3O4@PDA@COF at 25 °C.

Analytes	Pseudo-First-Order			Pseudo-Second-Order		
	*k*_1_	*q_e_* (mg g^−1^)	R^2^	*k*_2_	*q_e_* (mg g^−1^)	R^2^
MBF	0.0232	2.30	0.9939	0.0243	10.11	0.9998
LOM	0.0273	1.39	0.9856	0.0497	10.04	1
SPX	0.0248	1.88	0.9952	0.0328	10.09	0.9999

**Table 2:** Validation parameters of the MSPE-HPLC-UV method in a chicken sample.

Analytes	Linear Range (μg·kg^−1^)	R^2^	LOD (μg·kg^−1^)	LOQ (μg·kg^−1^)	Intraday RSD (%) (*n* = 3)	Interday RSD (%) (*n* = 3)
MBF	1–250	0.9938	0.7	2.5	3.9	5.4
LOM	1–250	0.9956	0.5	1.9	4.6	6.2
SPX	1–250	0.9963	0.5	1.7	6.1	7.8

**Table 3:** FQ analytical results in chicken samples (*n* = 3).

Analytes	Added (μg·kg^−1^)	Found (μg·kg^−1^)	Recovery (%)	RSD (%)
MBF	0	N.D. ^1^		
	5	4.7	94.0	4.8
	50	42.1	84.2	7.4
	100	105.6	105.6	5.1
LOM	0	N.D.		
	5	4.8	96.0	6.9
	50	51.2	102.4	7.8
	100	97.4	97.4	5.4
SPX	0	N.D.		
	5	4.4	88.0	7.4
	50	48.9	97.8	5.3
	100	106.7	106.7	6.9

^1^ N.D.: not detected.

**Table 4:** Comparison of the developed method with other reported methods.

Adsorbent	Method	Adsorbent Amount	Adsorption Time	Samples	LODs	Ref.
MMON-SO_3_H-NH_2_	MSPE	3 mg	6 min	chicken, beef, pork	0.05–4.5 μg·L^−1^	[34]
Fe_3_O_4_@COF(TpBD)	MSPE	20 mg	30 min	pork, chicken and bovine	0.1–1.0 μg·kg^−1^	[31]
MIM/C_3_N_4_	SPE	30 mg	60 min	chicken	0.2–0.8 ng·g^−1^	[35]
Fe_3_O_4_@LDH/ZIF	MSPE	12 mg	40 min	fish, pork, chicken, bullfrog, and beef	0.1–0.6 μg·kg^−1^	[3]
imprinted MOF	SPE	4 mg	30 min	pork	0.03–0.12 μg·kg^−1^	[36]
Fe_3_O_4_@PDA@COF	MSPE	10 mg	30 min	chicken	0.5–0.7 μg·kg^−1^	This method

## Data Availability

The original contributions presented in the study are included in the article and Supplementary Materials.

## Supplementary Materials

The following supporting information can be downloaded at https://www.mdpi.com/article/10.3390/molecules31061025/s1, Figure S1: Chemical structures of target analytes; Figure S2: Small angle XRD patterns of Fe_3_O_4_@PDA@COF; Figure S3: Reusability of Fe_3_O_4_@PDA@COF for the adsorption of FQs; Section S1: Instrumental Operating Conditions.

## Abbreviations

The following abbreviations are used in this manuscript:

FQs	Fluoroquinolones
MSPE	Magnetic solid phase extraction
COFs	Covalent organic frameworks
TAPT	4,4′,4″-(1,3,5-Triazine-2,4,6-triyl)trianiline
TFTA	2,3,5,6-Tetrafluorotelephtal aldehyde
LOM	Lomefloxacin hydrochloride
SPX	Sparfloxacin
MFB	Marbofloxacin
ACN	Acetonitrile
MeOH	Methanol
FA	Formic acid
DMF	N,N-dimethylformamide
XRD	X-ray diffraction
FT-IR	Fourier transform infrared spectroscopy
TEM	Transmission electron microscopy
PDA	Polydopamine
RSD	Relative standard deviations
