# Supplementary material for: Fabrication of Fluorinated Magnetic Covalent Organic Frameworks for the Extraction of Fluoroquinolone Residues Coupled with HPLC Detection
Source: Molecules. 2026 Mar 19;31(6):1025. doi: 10.3390/molecules31061025 (PMC13029347; doi:10.3390/molecules31061025)
Supplement: Supplementary file 1 [file molecules-31-01025-s001.zip › molecules-4162955-supplementary.pdf]

Supplementary Materials for

Fabrication of fluorinated magnetic covalent organic frame-works for the extraction of fluoroquinolone residues coupled with HPLC detection

Jichao Liu*, Xiuzhuang Li*, Jiaojiao Y

College of Petrochemical Engineering, Lanzhou Petrochemical University of Vocational Technology, Lanzhou 730060, China

* Correspondence: liujichao202115@163.com, lixiuzh03@163.com

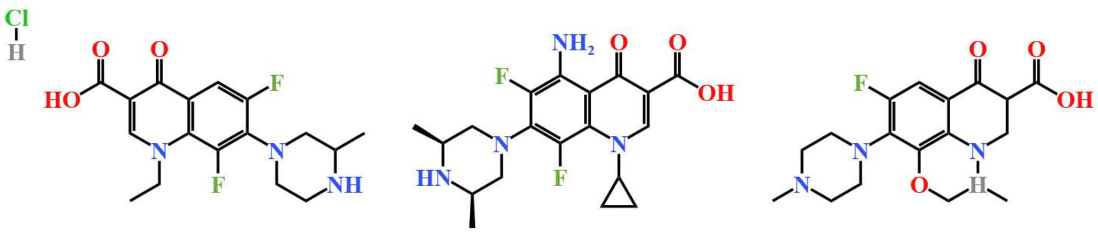

lomefloxacin hydrochloride (LOM) **sparfloxacin (SPX)** **marbofloxacin (MFB)**

Supplementary Figure S1. Chemical structures of target analytes

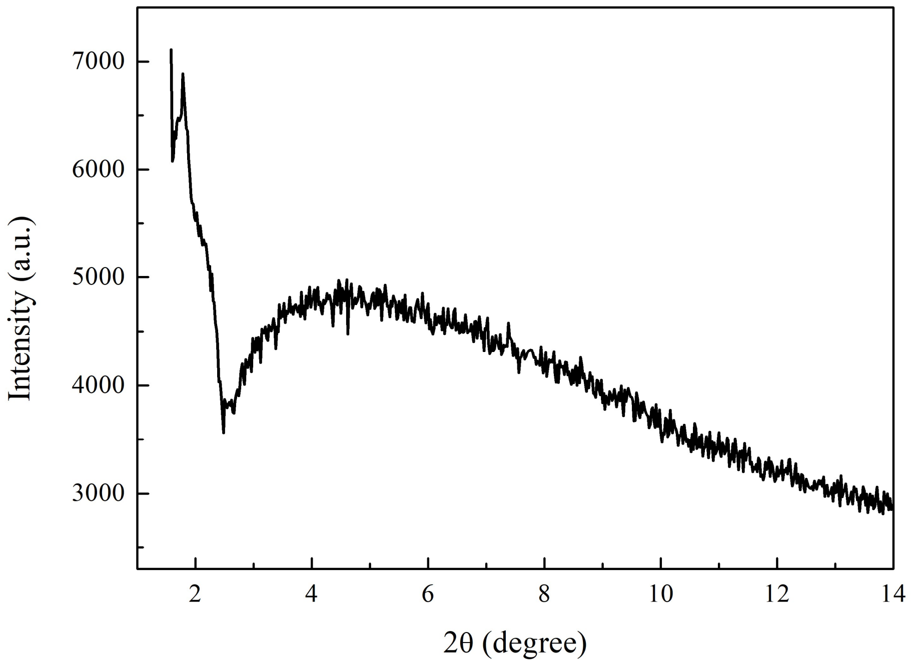

Supplementary Figure S2. Small angle XRD patterns of Fe_3O_4@PDA@COF

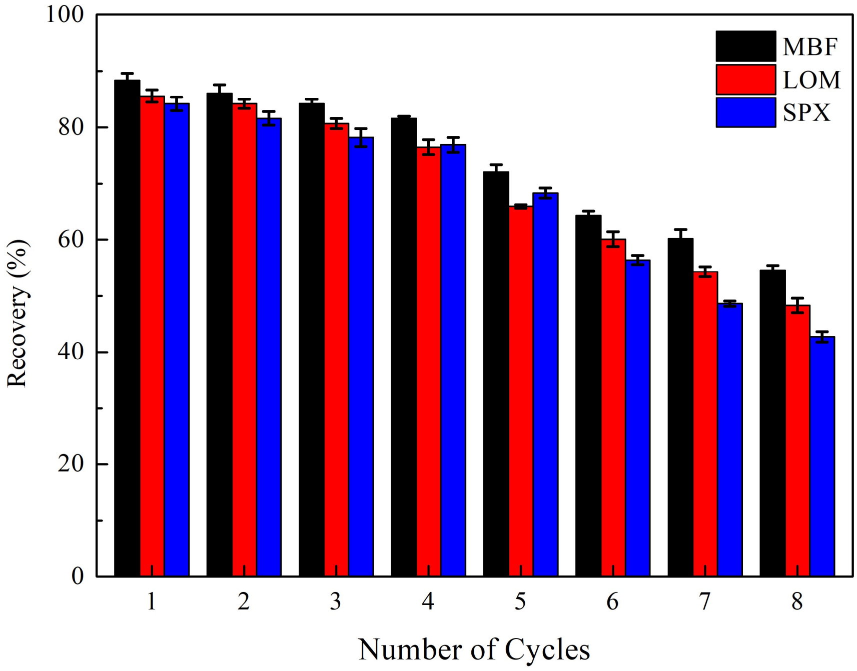

Supplementary Figure S3. Reusability of Fe_3O_4@PDA@COF for the adsorption of FQs

Section S1. Instrumental Operating Conditions

XRD: Data were collected over a 2θ range of 1.5–80° at a scanning rate of $2°\cdot min^{-1}$ with a step size of 0.02°.

FT-IR: The samples were ground with spectroscopic grade KBr and pressed into transparent pellets. Spectra were collected in the range of 4000–400 cm^{-1} at a resolution of 4 cm^{-1} with 32 scans accumulated.

N_2 adsorption–desorption curve: Prior to N_2 adsorption–desorption analysis, the samples were degassed for 6 h under vacuum. The measurements were performed at 77 K over a relative pressure (P/P_0) range of 0.01–0.99.

Magnetic hysteresis loops: The measurement conditions are at room temperature over a magnetic field range of -20,000 to +20,000 Oe.
